# Mediators of Inflammation-Induced Bone Damage in Arthritis and Their Control by Herbal Products

**DOI:** 10.1155/2013/518094

**Published:** 2013-02-07

**Authors:** Siddaraju M. Nanjundaiah, Brian Astry, Kamal D. Moudgil

**Affiliations:** ^1^Department of Microbiology and Immunology, University of Maryland School of Medicine, Baltimore, MD 21201, USA; ^2^Division of Rheumatology, Department of Medicine, University of Maryland School of Medicine, Baltimore, MD 21201, USA

## Abstract

Rheumatoid arthritis (RA) is an autoimmune disease characterized by chronic inflammation of the synovial joints leading to bone and cartilage damage. Untreated inflammatory arthritis can result in severe deformities and disability. The use of anti-inflammatory agents and biologics has been the mainstay of treatment of RA. However, the prolonged use of such agents may lead to severe adverse reactions. In addition, many of these drugs are quite expensive. These limitations have necessitated the search for newer therapeutic agents for RA. Natural plant products offer a promising resource for potential antiarthritic agents. We describe here the cellular and soluble mediators of inflammation-induced bone damage (osteoimmunology) in arthritis. We also elaborate upon various herbal products that possess antiarthritic activity, particularly mentioning the specific target molecules. As the use of natural product supplements by RA patients is increasing, this paper presents timely and useful information about the mechanism of action of promising herbal products that can inhibit the progression of inflammation and bone damage in the course of arthritis.

## 1. Introduction

**Figure 1:**
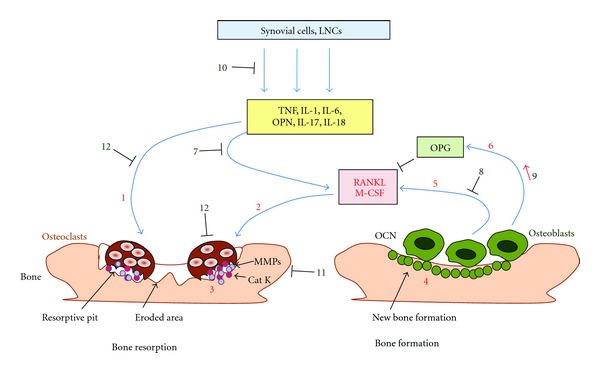
Bone remodeling involves the balance between osteoclast and osteoblast activity, and this balance can be influenced by herbal products. Osteoclasts activated by proinflammatory cytokines (no. 1) and RANKL/M-CSF (no. 2) initiate bone resorption by releasing catalytic enzymes like Cat K and MMPs (no. 3) in a resorptive pit formed on the bone surface. Osteoblasts facilitate bone formation by laying down a matrix, which subsequently is mineralized (no. 4). Osteoblasts produce RANKL (no. 5), which initiates osteoclastogenesis, while OPG (no. 6) inhibits RANKL. Natural products can afford protection against bone damage by (a) inhibiting RANKL production by activated T cells (no. 7) and osteoblasts (no. 8), (b) increasing the production of OPG (no. 9), which in turn keeps RANKL in check, (c) suppressing the proinflammatory cytokines (no. 10), (d) inhibiting production of Cat K and MMPs (no. 11) as well as their activity, and (e) inhibiting osteoclast formation (no. 12). The above-mentioned numbers 1–12 in parenthesis correspond to the numbers in the figure (⊣: inhibition) (Cat K, Cathepsin K; GM-CSF, granulocyte-macrophage colony-stimulating factor; IL, interleukin; LNCs, lymph node cells; M-CSF, macrophage colony-stimulating factor; MMPs, matrix metalloproteases; OCN, osteocalcin; OPN, osteopontin; OPG, osteoprotegerin; RANKL, receptor activator of NF-*κ*B ligand; TNF, tumor necrosis factor-*α*).

Rheumatoid arthritis (RA) is a chronic debilitating disease of autoimmune origin. Inflammation of the synovial lining leading to pannus formation is a characteristic feature of the disease. Untreated RA progresses to bone and cartilage damage in the joints leading to deformities and disability. Over the past decade, it has increasingly been recognized that inflammation can induce bone damage and that the two processes are linked via common mediators. These mediators include receptor activator of NF-*κ*B ligand (RANKL) and its receptor RANK, proinflammatory cytokines (e.g., tumor necrosis factor-*α* (TNF-*α*), interleukin 1 (IL-1), IL-6, IL-17, and IL-18), and matrix-degrading enzymes (e.g., matrix metalloproteases (MMPs) and cathepsin K (Cat K)) (Figure 1). The term “osteoimmunology” has been coined to highlight the above-mentioned interplay between inflammation and bone damage driven via various common immune mediators [1]. Bone remodeling refers to the intertwined processes of bone formation and bone resorption. Defined cell types, including osteoblasts, osteoclasts, synovial fibroblasts, and T helper cells, are involved in these processes. A variety of anti-inflammatory agents and other potent drugs are being used for the treatment of RA. However, these drugs are expensive and their prolonged use is associated with severe adverse reactions. For this reason, there is a growing need for newer therapeutic agents that are effective yet safe and less expensive. Natural plant products offer promising therapeutic agents in this regard. In this paper, we describe the basic pathophysiology of inflammation-induced bone damage, including detailed information on the cellular and soluble mediators involved in this process (Figure 1). In addition, we elaborate upon various natural plant products that show antiarthritic activity in animal models of arthritis, highlighting specific molecules and pathways targeted by these products (Table 1).

## 2. The Pathogenesis of Arthritic Inflammation

**Figure 2:**
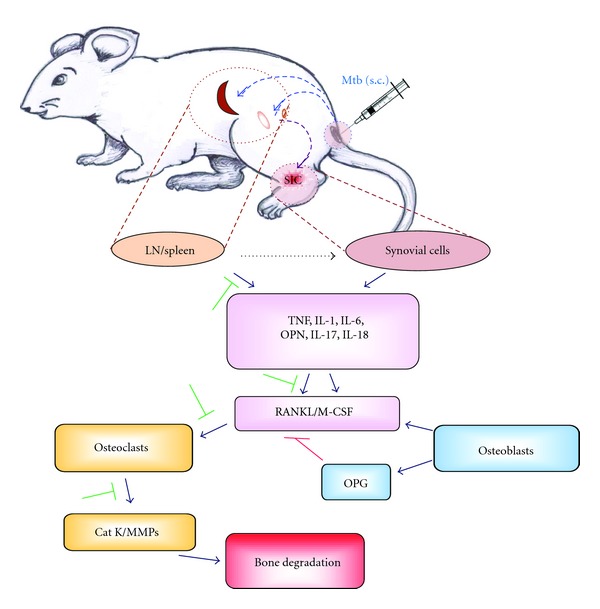
Arthritic bone damage and its modulation by herbal CAM. Adjuvant arthritis (AA) can be induced in Lewis rats by injection of heat-killed *M. tuberculosis* H37Ra (Mtb) at the base of the tail. The microbial antigens are then taken up by antigen-presenting cells (APCs) and then transported to the regional draining lymph nodes and spleen. APCs process antigens and then present them to antigen-specific T cells, which undergo activation and proliferation. These antigen-primed T cells then migrate into the target organ, the joints, and release proinflammatory cytokines locally leading to arthritic inflammation. These cytokines also stimulate the production of RANKL, which activates osteoclasts producing Cat K and MMPs resulting in bone damage (⊣: inhibition) (Cat K, Cathepsin K; IL, interleukin; LN, lymph node; M-CSF, macrophage colony-stimulating factor; MMPs, matrix metalloproteases; Mtb, *M. tuberculosis* H37Ra; OPN, osteopontin; OPG, osteoprotegerin; RANKL, receptor activator of NF-*κ*B ligand; s.c., subcutaneous; TNF, tumor necrosis factor-*α*).

Animal models of RA have extensively been used for studying the pathogenesis of the disease as well as for testing new antiarthritic agents. The rodent models of human RA belong to two broad categories, experimentally-induced and spontaneously-induced arthritis [2, 3]. Among the former, adjuvant arthritis (AA) and collagen-induced arthritis (CIA) represent two of the well-studied experimental models. Using the AA model as the prototype, we have elaborated in brief the main events leading to the disease process in arthritis. AA can be induced by immunization of Lewis (RT.1^l^) rats with heat-killed *Mycobacterium tuberculosis *(Mtb) (H37Ra) [4]. The paw inflammation appears after 8–10 days of Mtb injection, attains peak between 16–18 days, and then undergoes a spontaneous, gradual recovery in the subsequent 12–15 days. Following Mtb injection at the base of the tail, microbial antigens, including mycobacterial hsp65 (Bhsp65), are taken up and then transported through the local lymphatics into the regional draining lymph nodes, where antigen-presenting cells (APCs) process and present these antigens to naive T cells (Figure 2). This results in the activation and proliferation of antigen-specific T cells. These antigen-primed T cells then migrate into the target organ, the joints, via circulation. This leads to the initiation of arthritic inflammation [2, 3]. The coordinated interplay of cytokines, chemokines, and other mediators of inflammation then helps in the propagation of arthritic inflammation. This is followed by spontaneous regression of inflammation. Uncontrolled inflammation leads to damage to the bone and cartilage within the joints. Inflammation-induced bone damage involves mediators that are shared between the immune system and the bone remodeling system, and this interplay has been termed “osteoimmunology” [1]. The mechanisms involved in bone remodeling and their alterations during the course of arthritis are elaborated below.

## 3. Cellular Participants in Bone Remodeling

### 3.1. Osteoblasts

Osteoblasts are mononuclear cells that are responsible for bone formation, and bone-forming osteoblasts comprise approximately 5% of bone cells. The main function of the osteoblast is the formation of new bone and the regulation of bone resorption [46, 47]. Osteoblasts are derived from osteoprogenitor cells that are located in the periosteum and the bone marrow and express the master regulator transcription factor, core binding factor alpha (Cbfa)-1/Runt-related transcription factor (Runx)-2. The hematopoietic system provides the cytokines and growth factors required for bone cell regeneration. Mesenchymal stem cells expressing high levels of hormones and cytokine receptors, such as prostaglandin (PG), parathyroid hormone (PTH), insulin-like-growth-factor- (IGF-) 1, IL-1, and transforming-growth-factor- (TGF-) *β* receptors, become osteoprogenitors [48]. Wingless (Wnt) and bone morphogenic proteins (BMPs) drive these early events. Runx2 is the master transcription regulator for the osteoblast. Osterix is another transcription factor that is essential for osteoblast differentiation; it interacts with nuclear factor of activated T-cells (NFAT) 2 and stimulates osteoblastogenesis and bone formation. Expression of osterix is regulated by BMP-2, N-cadherin, E-cadherin, and IGF-1 [48]. The cells then stop proliferation, begin to secrete noncollagenous matrix proteins and type 1 collagen, and express alkaline phosphatase (ALP). Osteoblast maturation is a stepwise process involving entry into the osteoblastic lineage, cell proliferation, bone matrix deposition, matrix maturation, and bone mineralization. Osteoblasts may become osteocytes or chondrocytes, or undergo apoptosis. Osteoblasts communicate with osteocytes to receive mechanotransduction signals through gap junctional connexins. PTH and IGF-1 regulate osteoblast activity. Wnt-*β*-catenin signaling is another critical pathway for bone remodeling [49, 50]. However, due to the limited scope of this paper, this signaling pathway will not be discussed further.

### 3.2. Osteoclasts

Osteoclasts not only play a critical role in skeletal development and maintenance, but also mediate the pathogenesis of bone-related diseases such as RA and osteoporosis. Osteoclasts are large multinuclear cells derived from hematopoietic stem cells [51]. Their differentiation pathway is common to that of macrophages and dendritic cells (DCs). However, the exposure of a promyeloid precursor to RANKL and macrophage colony-stimulating factor (M-CSF) promotes osteoclast formation [52, 53]. These two factors are produced by bone marrow stromal cells, osteoblasts, and activated T cells [54, 55]. In fact, T cells and their products serve as key regulators of osteoblast and osteoclast formation, survival, and function. Subsequent activation of a mature, multinucleated osteoclast by RANK-RANKL interaction involves structural changes that prepare it to cause bone resorption. Such changes include the rearrangement of the actin cytoskeleton to form a tight junction between the bone surface and the basal membrane resulting in a sealed compartment. Osteoclasts attach to bone via filamentous actin and *α*v*β*3 integrin [56]. Carbonic anhydrase II (CAII) generates H^+^ and HCO_3_ from CO_2_ and H_2_O. An osteoclast-specific H^+^-ATPase pump and the chloride channel (CLCN)7 transport protons and chloride ions, respectively, across the ruffled border into resorption lacunae [57]. The acidic environment promotes dissolution of bone mineral to release Ca^2+^, HPO_4_^3−^, and H_2_O. The organic matrix is degraded by Cat K [58]. The degradation products including collagen fragments and solubilized calcium and phosphate are processed within the osteoclast and released into the circulation. 

### 3.3. Osteocytes

Osteocytes comprise 90–95% of all adult bone cells and represent the third basic cell type in bone. They are derived from mature mineralizing osteoblasts that have become embedded in bone matrix [59]. Osteocyte cell bodies lie within the bone lacunae and their numerous dendritic processes ramify though networks of canaliculi. Osteocytes do not contain ALP, but produce osteopontin (OPN) along with other BMPs. Mechanical stresses and localized microdamage control osteocyte signaling pathways that result in the release of cytokines and chemotactic signals, or they may induce osteocyte apoptosis. Increased mechanical stress stimulates local osteoblastic bone formation, whereas reduced loading or microdamage results in osteoclastic bone resorption [60].

## 4. Soluble Mediators and Their Receptors Involved in Bone Remodeling

### 4.1. Receptor Activator of Nuclear Factor Kappa B Ligand (RANKL)

RANKL is also known as tumor necrosis factor ligand superfamily member 11 (TNFSF11), TNF-related activation-induced cytokine (TRANCE), osteoprotegerin ligand (OPGL), and osteoclast differentiation factor (ODF). Together with M-CSF, RANKL is essential for the activation, maturation, and survival of osteoclasts. RANKL is highly expressed in osteoblasts [61]. RANKL expression can be upregulated by bone-resorbing proinflammatory cytokines such as IL-1, IL-6, IL-17, and TNF-*α*. RANKL is a critical requirement for osteoclastogenesis. The absence of any molecule downstream of RANK-RANKL pathway interferes with osteoclastogenesis. NFAT2 is the most distal target, and ectopic expression of NFAT2 can induce differentiation of osteoclast precursors without the need for signaling via RANKL. RANKL can also act directly on T cells leading to stimulation of c-Jun amino-terminal kinase (JNK) activity [62] and increased survival of activated T cells [63].

### 4.2. Receptor Activation of NF-*κ*B (RANK)

Other names of RANK are TRANCE-R, ODFR, or TNFRSF11A. RANK is the cognate receptor for RANKL and is a member of TNF-R superfamily. RANK is expressed on the surface of osteoclast progenitors, mature osteoclasts, and chondrocytes [63–65]. The binding of RANKL to RANK results in osteoclastogenesis from progenitor cells and the activation of mature osteoclasts [65, 66]. RANKL-RANK interaction sends signals into the cells through TNFR-associated factors (TRAFs). Cellular sarcoma (c-Src) and casitas B-lineage lymphoma (Cbl) proteins associate with the cytoplasmic tail of RANK and relay the signal to downstream pathways such as NF-*κ*B, JNK/stress-activated protein kinase (SAPK), p38, and Akt/protein Kinase B (PKB), which then regulate bone resorption [67]. TRAF6 is a critical factor involved in the activation of mature osteoclasts. Other TRAFs may partially substitute for the loss of TRAF6 during osteoclast development. Interferon gamma (IFN-*γ*) can inhibit RANKL-mediated osteoclastogenesis presumably via ubiquitination and proteolytic degradation of TRAF6 [61]. 

### 4.3. Osteoprotegerin (OPG)

OPG is also known as osteoclastogenesis inhibitory factor (OCIF). It is a decoy receptor for RANKL and competes with RANK for the same ligand. OPG is a soluble secreted protein with homology to members of the TNF receptor family [68, 69]. OPG inhibits the maturation and activation of osteoclasts [52]. High systemic levels of OPG may cause osteopetrosis [70]. Besides binding RANKL, OPG can bind to the TNF family molecule, TNF-related apoptosis-inducing ligand (TRAIL), but the binding is much weaker than that to RANKL. Furthermore, TRAIL can block the antiosteoclastogenic activity of OPG [71]. The complex system of osteoclast-regulated bone remodeling is critically dependent on the RANKL-RANK-OPG axis. Although RANKL is also expressed in many other tissues other than the bone, osteoclast development is restricted to the bone microenvironment suggesting the involvement of another tissue specific factor for this restricted effect.

### 4.4. Macrophage Colony-Stimulating Factor (M-CSF)

M-CSF is mainly produced by the mature osteoblasts, but it can also be produced by chondrocytes and synovial fibroblasts in response to the proinflammatory cytokines IL-1 and TNF-*α*. M-CSF and RANKL are the major stimuli for inducing proliferation, survival, and differentiation of early precursors of osteoclasts. M-CSF binds to its receptor c-Fms (colony-stimulating factor 1 receptor), a transmembrane tyrosine kinase-receptor, on the surface of osteoclast precursors [53], and M-CSF-c-Fms interaction initiates a series of phosphorylation events resulting in the formation of a multimeric complex that is involved in cytoskeletal reorganization and cell motility [72]. 

### 4.5. Osteocalcin (OCN)

OCN is a major noncollagenous matrix protein of bone secreted solely by osteoblasts, which constitutes 2% of the total protein content in bone. It is distributed in cement lines of both cortical and trabecular bone. OCN is crucial in regulating osteoblast activity and binding of hydroxyapatite [73]. Osteocalcin is pro-osteoblastic or bone-building in nature. Activation of RUNX2 and/or osterix leads to increased expression of osteoblast-specific genes, such as those encoding ALP and osteocalcin. Serum osteocalcin may serve as an index of bone turnover in active RA [74].

### 4.6. Osteopontin (OPN)

OPN is one of the abundant noncollagenous proteins in bone. Osteopontin-deficient mice are resistant to ovariectomy-induced bone resorption [75]. OPN can enhance the osteoclastic bone resorption and suppress the osteoblastic bone formation [75]. OPN also facilitates angiogenesis, accumulation of osteoclasts, and resorption of bone in ectopic bone [76]. Endogenous OPN is produced in RA synovial fibroblasts (RASFs) and it increases the production of IL-17 in T cells [41]. OPN overexpression enhances the production of IL-6 in RA [77].

### 4.7. Insulin-Like Growth Factor 1 (IGF1)

IGF1 mediates bone and cartilage degradation. Increased levels of IGF and IGF-binding protein (IGFBP)-3 are found in the synovial fluid of RA patients. IGF1 and endothelin-1 (ET1), which bind to the receptor tyrosine kinases IGF1R and G-protein-coupled receptor ETA receptor, respectively, have both been shown to activate the mitogen-activated protein kinase (MAPK) pathway in osteoblasts.

## 5. Cytokines: Immune Effector Molecules Upstream of the Mediators of Bone Damage

### 5.1. Interleukin-17 (IL-17)

Since the discovery of IL-17, several studies have validated the role of this cytokine in inflammatory and autoimmune diseases. The IL-17 family of cytokines contains several isoforms. The first discovered and most extensively studied is IL-17A that simply is referred to as IL-17. It is released by many different cell types including CD4^+^ T cells (Th17 cells), *γδ* T cells, natural killer T (NKT) cells, NK cells, lymphoid tissue inducer (LTi) cells, neutrophils, and mast cells [78, 79]. IL-17 is a proinflammatory cytokine that has been shown to be associated with several autoimmune diseases, including RA [80]. In the case of autoimmune inflammation, IL-17 stimulates the production of proinflammatory cytokines, chemokines, and vascular endothelial growth factor (VEGF), leading to inflammatory cell infiltration into the target organs [78]. IL-17 also contributes to the bone pathology in RA by upregulation of RANK on the surface of osteoclasts [81], and the stimulation of monocytes to produce TNF-*α* resulting in increased RANKL expression [82]. OPN has been shown to promote Th17 differentiation by inducing IL-17 production by CD4^+^ T cells through epigenetic modification of the *IL-17A* locus. This OPN-induced IL-17 production has been observed in CD4^+^ T cells derived from RA patients [41, 83]. These effects lead to osteoclastogenesis and ultimately bone resorption in RA; however, OPG and infliximab (anti-TNF-*α* monoclonal antibody (mab)) can inhibit the effect of IL-17 on osteoclastogenesis [82].

### 5.2. Interleukin-18 (IL-18)

IL-18 is a member of the IL-1 family of cytokines. It is first synthesized in a pro-IL-18 form and must be cleaved by caspase 1 into the active form before being released. IL-18 is produced primarily by macrophages, but other sources include fibroblasts, endothelial cells, DCs, osteoblasts, and neutrophils [84–86]. In inflammatory disease states like RA, IL-18 is significantly increased in the inflamed tissue [84]. In RA, TNF-*α* stimulates synovial fibroblasts to produce IL-18, which can synergize with other cytokines to activate macrophages to produce TNF-*α* and IL-1*β*, leading to a positive feedback loop escalating joint inflammation [86]. The bone pathology associated with RA can be attributed to increased osteoclastogenesis. IL-18 upregulates soluble RANKL production and RANKL expression on the surface of activated T cells in the synovium of an arthritic joint. This upregulation leads to increased osteoclast formation and bone resorption activity by the osteoclasts [87]. Although IL-18 has no significant effect on the expression of OPG, granulocyte-macrophage colony-stimulating factor (GM-CSF), or M-CSF in activated T cells, it upregulates OPG expression by stromal and osteoblastic cells, indicating a negative feedback mechanism used by osteoblasts to control osteoclastogenesis [87, 88]. Furthermore, IL-18 inhibits osteoclast formation via GM-CSF when M-CSF is absent from the culture environment [89]. Further, IL-18 can synergize with IL-12 in the production of IFN-*γ* [90], indicating that IL-18 has a dual role in the regulation of osteoclastogenesis and bone resorption.

### 5.3. Tumor Necrosis Factor-*α* (TNF-*α*)/IL-1/IL-6

For decades, IL-1 and TNF-*α* have been implicated in the differentiation and activation of osteoclast cells. As the field of osteoimmunology expands, the mechanisms associated with these proinflammatory cytokines are becoming better understood. TNF-*α* is primarily released by activated macrophages and it signals through two types of TNF receptors (TNFRs), one that causes apoptosis and a second that is activating in nature. IL-1 is secreted by many cell types and is a pleiotropic cytokine [91]. IL-1*β* and TNF-*α* are capable of stimulating membrane-bound RANKL in activated T cells resulting in osteoclastogenesis and increased bone resorption activity [87]. IL-1*β* appears to have little effect on osteoclastogenesis in the absence of M-CSF and RANKL, but in their presence IL-1*β* increases osteoclastogenesis 4-fold [92]. In addition to a RANKL-dependent pathway, TNF-*α* can stimulate osteoclast differentiation in the presence of M-CSF through TNFR1 and TNFR2 independent of RANKL [93]. However, RANKL-independent TNF-*α*-induced osteoclast differentiation does not result in substantial bone loss because signaling through TRAF6 has been shown to be important for osteoclast activation [94, 95]. As a result, TNF-*α*-differentiated osteoclasts require IL-1*β* for activation and bone resorption [94]. In mouse embryonic fibroblasts, IL-1*β*, TNF-*α*, and IL-6 stimulate the production of IL-6 and other IL-6 cytokine family members. IL-6 signals through signal transducer and activator of transcription (STAT)3 and stimulates IL-1 and RANKL production. This indicates an amplification loop associated with IL-1*β*, TNF-*α*, and IL-6 signaling leading to increased osteoclastogenesis and bone resorption in autoimmune arthritis [91, 96]. Furthermore, IL-1*β* and TNF-*α* are unable to stimulate the production of M-CSF, GM-CSF, or OPG in resting or activated T cells [87].

 IL-6 is a glycoprotein (gp)130 family cytokine that is produced by macrophages, DCs, endothelial cells, and T cells. It is a proinflammatory cytokine that can help differentiate T helper (Th) 17 cells, which play a role in bone damage in pathological states like RA [1, 78, 94]. Functional studies have indicated that IL-6 does not directly stimulate osteoclast cells, but rather stimulates other cell types like osteoblasts, stromal cells, and fibroblasts to produce RANKL, leading to osteoclastogenesis [91, 96]. The importance of IL-6 in stimulating bone loss has clinical relevance. RA patients that received the anti-IL-6 monoclonal antibody, tocilizumab, showed a significant increase of OPG expression when compared with patients on methotrexate [97]. On the other hand, when IL-6 and soluble IL-6 receptor (sIL-6R) are combined, there is an increase in RANKL and OPG expression associated with a decrease in RANK expression [91]. This is similarly seen in chondrocyte culture with an additional decrease of M-CSF production leading to decreased osteoclastogenesis [98]. Although this may appear to indicate a dual role of IL-6, as stated above, clinical evaluation of anti-IL-6 biologics shows decreased bone loss.

## 6. Effector Molecules Downstream of the Mediators of Bone Remodeling

### 6.1. Cathepsin K (Cat K)

Cat K is a papain-like cysteine protease that can degrade collagen, and it is mainly expressed in osteoclasts. Cat K is synthesized as a proenzyme (mol. wt. 37 kDa) and secreted into the resorptive pit. Upon activation by autocatalytic cleavage, it is transformed into its active form (mol. wt. ~27 kDa). Cat K is the major collagenase in bone that mediates osteoclastic bone resorption. It has the ability to degrade the extracellular matrix (ECM) proteins such as collagen, elastin, and osteonectin [99]. Many factors can stimulate the production of Cat K from osteoclast such as RANKL, NFAT, TNF-*α*, IL-1*β*, and IL-17 [58, 100].

### 6.2. Matrix Metalloproteases (MMPs)

MMPs are a group of zinc-dependent endopeptidases that can degrade the ECM, particularly the proteoglycan aggrecan and type II collagen. MMP-1, -2, -8, -13, and -14 are collagenolytic enzymes produced by chondrocytes as well as synoviocytes. In health, MMPs are produced in small quantities for connective tissue remodeling. However, in diseases like arthritis, MMPs are produced in excess leading to osteoclastic resorption and degradation of a number of noncollagenous proteins of the bone matrix [101]. Among the MMPs, MMP-2 (gelatinase A) and MMP-9 (gelatinase B) are especially important in collagen degradation [102]. MMPs can also modify cell-cell and cell-ECM interactions. MMP-9 is expressed by osteoclasts and it is required for osteoclast migration [103]. It is one of the physiological modulators of RANKL activity, which has been shown to play an important role in bone remodeling [104]. MMP-9 facilitates RANKL-induced osteoclastogenesis independent of NFATc1 signaling [104]. MMP-13 is involved in bone resorption and osteoclast differentiation. MMPs expressed by the osteoclast include MMP-9, -10, -12, and -14. The expression/activity of MMPs can be modulated by the proinflammatory cytokines (e.g., IL-1*β*, TNF-*α*, IL-6, and IL-17) that are expressed in the synovial tissue and synovial fluid in arthritic joints [105, 106]. 

### 6.3. Tartrate-Resistant Acid Phosphatase (TRAP)

TRAP is a glycosylated metallophosphoesterase participating in osteoclast-mediated bone turnover [107]. It has a molecular weight of approximately 35 kDa and has optimal activity in acidic conditions. It can be differentiated from other mammalian acid phosphatases by its molecular weight, characteristic purple color, and resistance to inhibition by tartrate. TRAP is synthesized as a latent proenzyme and it is activated by Cat K and other MMPs [108]. TRAP is highly expressed by osteoclasts and is localized within the ruffled border area as well as the lysosomes and vesicles [107]. TRAP dephosphorylates osteopontin and allows osteoclast migration and bone resorption. TRAP also helps in osteopontin dephosphorylation, and generation of reactive oxygen species (ROS) [109].

### 6.4. Mediators of Bone Remodeling and Their Network

Proinflammatory cytokines such as IL-1*β*, TNF-*α*, IL-6, IL-17, and IL-18 produced by the immune cells in the lymph node and in the synovial-infiltrating cells (SICs) in the joints induce osteoclastogenesis directly or via RANKL, initiating bone resorption by releasing catalytic enzymes like Cat K and MMPs in a resorptive pit formed on the bone surface as described above (Figure 1). On the other hand, osteoblasts also regulate osteoclastic activity by expressing RANKL and OPG. In this context, natural products can influence bone damage by (a) inhibiting RANKL production by activated T cells and osteoblasts, (b) increasing the production of OPG, which in turn keeps RANKL in check, (c) suppressing the proinflammatory cytokines, (d) inhibiting the production of Cat K and MMPs as well as their activity, and (e) directly suppressing osteoclast formation.

## 7. Practical Aspects of the Study of the Mediators of Bone Damage

### 7.1. Therapeutic Approaches Exploiting the RANKL-RANK-OPG Axis

RANKL signaling can be inhibited using OPG, OPG-mimetics, RANK fragments, or monoclonal antibodies against RANKL or RANK. OPG can inhibit bone resorption. Treatment with monoclonal antibody AMG162 against RANKL inhibits bone resorption and increases bone mineral density [110]. Although AMG162 appears safe for in vivo use in humans, there are concerns relating to its long-term side effects.

### 7.2. Dissociation between Inflammation and Bone Damage in Arthritis

The inflammatory process in RA drives bone destruction. Therefore, controlling inflammation helps prevent bone damage. However, from therapeutics point of view, not all antiarthritic agents affect both processes equally. Certain drugs/targets may have a more prominent role in bone damage than in joint inflammation, or the reverse. Clinical trials in RA patients have suggested the uncoupling of bone erosion and joint inflammation [111]. Smolen and colleagues [112] showed that there was a radiographic benefit after combination treatment with infliximab and methotrexate (MTX) in RA patients with no clinical improvement. In another study, overexpression of OPG suppressed bone erosion in animals with CIA [113], in IL-17-induced arthritis [114], and in TNF-*α*-transgenic mice, but without a significant effect on the inflammation component of arthritis. A similar uncoupling was observed in RANKL-deficient arthritic mice, where bone erosion was reduced, but synovitis and cartilage erosion were unaffected [115]. In a different study [116], the treatment of mice having CIA by adenoviral overexpression of IL-4 reduced cartilage and bone erosion along with downregulation of the RANKL/OPG balance, but without much effect on the total mass of the inflammatory synovial tissue. In this case, the inflammation persisted but was not destructive in nature [116]. In view of the above information, it is essential to search for novel antiarthritic agents that can inhibit both inflammation and bone damage in arthritis. Natural plant products offer a vast repertoire of agents that might possess such attributes.

### 7.3. The Practical Utility of Measuring Mediators of Bone Damage in Arthritis

The knowledge regarding the above-mentioned mediators of inflammation-induced bone damage has been used to assess bone damage during the course of arthritis. In experimental models of RA (e.g., AA and CIA), the serum, synovial fluid (SF), SIC, spleen adherent cells (SACs), and the draining lymph node cells (LNCs) of the arthritic animal can be tested for many of these markers, and the results then compared with those from control animals. In patients with arthritis, serum, SF, and synovial fluid mononuclear cells (SFMCs) are frequently tested for a similar set of markers. In addition, mechanistic aspects of bone damage can be tested in vitro, for example, using RAW 264.7 macrophage cells for osteoclastic activity, and MC3T3 cells for osteoblastic activity [117]. The bone damage-related markers can be tested in the specimen either as proteins (e.g., by ELISA, Multiplex, or Western blotting) or mRNA (by quantitative real-time PCR). The analysis of the results and their interpretation is easy for some markers but not that clear for others. It is somewhat difficult to categorize all the mediators of bone damage into two definitive categories of bone damage-promoting versus bone damage-protective markers, but for many of them the association with the disease process is quite clear. For example, RANKL, M-CSF, IL-17, IL-6, TNF-*α*, IL-1*β*, Cat K, TRAP, and some MMPs (MMP-2, -8, -9, -13, and -14) can easily be categorized as osteoclastic, that is, factors involved in bone resorption. On the contrary, OPG, OCN, IFN-*γ*, and IL-4 can be categorized as antiosteoclastogenic or bone-protective factors. A third group of markers such as IGF-1 and GM-CSF shows a dual role.

## 8. Herbal Products Used to Treat or Prevent Arthritis

Medicinal plants and their extracts have long been used as therapeutic agents for arthritis treatment in the traditional systems of medicine in many parts of the world [118–124]. In addition, medicinal plants have served as the source of several biologically active compounds that have formed the basis of development of new drugs by pharmaceutical companies. Many of the medicinal plants have been evaluated for their potential application in the treatment of arthritic inflammation and bone damage [120, 122, 125]. Investigators have employed several parameters to measure inflammation and tissue damage in arthritis. These include arthritic scores, cytokine profiles, chemokine profiles, bone remodeling molecules, histology, X-ray, bone histomorphometry, and micro CT. The effect of herbal products on arthritis can be tested and validated using the above parameters.

### 8.1. Antiarthritic Activity of Crude Plant Extracts

Crude aqueous or organic solvent extracts of traditional medicinal plants have been used for the treatment of arthritis; for example, (1) hexane extracts of *Cassia alata* leaves markedly reduced arthritis in rats with complete Freund's adjuvant (CFA)-induced arthritis. Cassia extract also showed protective effects against cartilage degradation in the knee joint [120]. (2) An extract prepared from the leaves of *Urtica dioica* has arthritis-inhibiting properties and it acts via inhibiting NF-*κ*B [121]. (3) The resinous exudate from the bark of *Commiphora* plant represents indigenous medicine used against arthritis [123]. (4) The dried leaf powder of *Salacia reticulata * (SRL) ameliorated collagen antibody-induced arthritis (CAIA) in mice. It suppressed the paw swelling, inflammatory cell infiltration, skeletal tissues damage, and osteoclast activation [122]. (5) An ethanol extract of *Trigonella foenum*-*graecum * (TFG) was tested against AA in rats [124]. It significantly reduced the paw volume and suppressed IL-1*α*, IL-1*β*, IL-2, IL-6, and TNF-*α* levels. In addition, it decreased the production of lipid peroxides (LPO) but increased the production of superoxide dismutase (SOD) and glutathione (GSH) in cartilage tissue [124]. (6) A water-extract of Dipsaci radix (DR), the dried root of *Dipsacus asperoides*, significantly suppressed the arthritic scores and serum levels of anti-CII IgG2a antibody, PGE(2), TNF-*α*, IL-1*β*, and IL-6 in mice with CIA [118]. (7) Similarly, *Morus bombycis* Koidzumi (MB) extract significantly decreased the clinical arthritis index and suppressed the expression of cytokines (TNF-*α*, IL-1*β*, IL-6), chemokines (MIP-1*α*, RANTES), and MMPs (MMP-1, MMP-3) in mice with CIA [119]. (8) Hydroalcoholic extract and ethyl acetate fraction from roots of *Hemidesmus indicus* showed potent antiarthritic activity in rats with AA. Specifically, there was a decrease in the paw edema, body weight, arthritic index, erythrocyte sedimentation rate (ESR), serum rheumatoid factor (RF), serum C-reactive protein (CRP), and serum nitrite levels [126]. (9) Hydroalcoholic extract of *Zingiber officinale* rhizomes (Ginger extract) showed an antiarthritic effect as tested in the rat CIA model. Ginger extract ameliorated the clinical scores, disease incidence, joint swelling, expression of IL-1*β*, IL-2, IL-6, and TNF-*α*, and cartilage destruction [127]. (10) Root extract from Astragalus (*Radix astragali*) reduced cellular accumulation, swelling and arthritic index of the joints, as well as serum levels of TNF-*α* and IL-1*β* in rats with AA [12]. (11) Methanolic extract of *Ruta graveolens* L. (Rutaceae) showed decreased edema formation, cellular infiltration and levels of CRP and ceruloplasmin in the rat AA model [128]. (12) The aqueous extract of *Strychnos potatorum* Linn seeds (SPE) and the whole seed powder (SPP) showed an antiarthritic effect in the AA model. This beneficial effect was validated by histopathological and radiological examination of arthritic paws of rats with AA [125]. (13) Green tea extract, a product of the dried leaves of *Camellia sinensis*, has been tested for antiarthritic activity, and the treatment of rats with AA by green tea extract showed reduced arthritic scores and IL-17 response, but increased production of immunoregulatory cytokine IL-10 [44]. (14) *Celastrus aculeatus* Merr. (Celastrus), a Chinese medicinal herb, has potent antiarthritic activity as tested in the rat AA model. Celastrus extract suppressed the level of proinflammatory cytokines (IL-17, IL-6, and IFN-*γ*), along with reduction in the expression of IL-6/IL-17-related transcription factor STAT3. It also suppressed MMP9 activity. In addition, phosphorylation of ERK was inhibited [10, 129]. Furthermore, Celastrus is effective in ameliorating immune-mediated bone damage by inhibiting RANKL expression and reducing the number of osteoclasts in the affected bones [11].

### 8.2. Antiarthritic Activity of Herbal Mixture/Formula

Representative examples are given below: (1) a combination of herbal extracts from *Trachelospermi caulis* (TC) and *Moutan cortex radicis* (MC) (TCMC) has an antiarthritic effect against CIA by suppressing the expression of various inflammatory mediators as well as the formation of osteoclasts, in part via inhibition of NF-*κ*B and AP-1 [130]. (2) Yunnan Baiyao, a Chinese herbal medicine, showed an antiarthritic effect against AA by regulating arachidonic acid metabolism in osteoblasts [131]. (3) RvCSd, an oriental herbal mixture, inhibited the production IL-1*β*, IL-2, IL-6, TNF-*α*, and MMP-1 and upregulated the anti-inflammatory cytokines IL-4, IL-10, and tissue inhibitor of metalloproteinase (TIMP)-1 in mice with CIA [132]. RvCSd treatment also reduced joint swelling, synovial hyperplasia, and cartilage destruction. (4) QFGJS is another Chinese herbal formula that showed a significant reduction of both paw swelling and proinflammatory cytokines (TNF-*α*, IL-1*β*, and IL-6) in AA [133]. (5) Tongbiling (TBL) is a Chinese herbal formula that has been used for treatment of RA. A water extract of TBL reduced paw inflammation, the serum levels of IL-1*β* and TNF-*α*, and the destruction of cartilage and bone in CIA by regulating the levels of MMP-2, -3, and -9 in the joints [134]. (6) Oral administration and intraperitoneal injection of Lingzhi and San Miao San (SMS) combination suppressed edema and hyperemia in the inflamed knees and also reduced the immune cell infiltration and erosion of joint cartilage in AA [135]. (7) Huo Luo Xiao Ling Dan (HLXL) and its modified versions have been used in traditional Chinese medicine for the treatment of pain and inflammation for many decades. HLXL-treated rats with AA showed decreased arthritis scores as well as the levels of proinflammatory cytokines (IL-1*β*, IL-6, IL-17, TNF-*α*) [136–138], chemokines (RANTES, MCP-1, MIP-1*α*, GRO/KC), and MMPs [139].

### 8.3. Antiarthritic Activity of Plant-Derived Phytochemicals-Flavonoids, Triterpenes, and Polyphenols

#### 8.3.1. Flavonoids

The mechanisms of action of some of the flavonoids with antiarthritic activity are outlined below: (1) 6-shogaol is one of the major compounds in the ginger rhizome. 6-Shogaol treatment reduced the concentration of soluble vascular cell adhesion molecule-1 (VCAM-1) in the blood, as well as the infiltration of leukocytes, lymphocytes, and monocytes/macrophages into the synovial cavity of the knee joint [30]. 6-Shogaol also protected morphological integrity of the cartilage lining the femur in CFA-induced monoarthritis of the knee joint of rats. (2) Naringin, a citrus flavanone, reduced arthritis as assessed clinically and histologically and afforded protection against interchondral joints damage in rats with CIA [32]. (3) A similar effect was observed with another citrus flavonoid, hesperidin, against CIA [33]. (4) Total flavonoid of orange (TFO) decreased paw thickness and improved pathological condition of ankle joint in rats with AA. TFO also suppressed elevated TNF-*α*, IL-1*β*, and PGE2 levels in serum and COX-2 expression in the synovial tissue [41]. (5) Genistein is an isoflavone, which suppressed the levels of IFN-*γ*, increased the production of IL-4, and normalized the Th1/Th2 balance in CIA [27]. Genistein also inhibited the proliferation of FLS in rats with CIA by inhibiting phosphorylation of ERK and downregulating tyrosine kinase of MAPK signal transduction pathway [28].

#### 8.3.2. Triterpenes

Examples of triterpenes possessing an antiarthritic attribute are as follows. (1) Lupeol, a pentacyclic triterpene isolated from the latex of *Calotropis gigantea, * showed potent antiarthritic activity as evaluated in the rat AA model. Lupeol ameliorated the paw swelling and reduced the levels of proinflammatory cytokines such as TNF-*α*, IL-1*β*, and IL-6 [29]. (2) Celastrol, a triterpene extracted from Celastrus, is a potent antiarthritic biomolecule. Both Celastrus extract and its bioactive component Celastrol suppressed the expression of proinflammatory cytokines (IL-17, IL-6, and IFN-*γ*), the transcription factor STAT3 for IL-6/IL-17, and the activity of matrix-degrading enzyme MMP-9. In addition, it inhibited the phosphorylation of ERK [10, 11, 140]. Celastrol also has a bone damage-protective effect. Celastrol inhibits osteoclastic activity via reducing RANKL production and suppressing the elevated RANKL/OPG ratio in rats with AA [11]. (3) *Tripterygium wilfordii* Hoog-derived trypterine reduced the paw swelling and bone destruction in AA. It also suppressed the expression of IL-1*β* and TNF-*α* in arthritic rats [141]. (4) Boswellic acid is a pentacyclic triterpene isolated from boswellia plants. It reduced leukocyte infiltration into the knee joint and the pleural cavity as observed in bovine-serum-albumin- (BSA-) induced arthritis in rabbits [21]. 3-Acetyl-11-keto-beta-boswellic acid (AKBA), which is well known for anti-inflammatory activity, also has antiarthritic activity. (5) Topical application of the polymeric nanomicelles of AKBA showed potent anti-inflammatory and antiarthritic activities [22].

#### 8.3.3. Polyphenols

Several polyphenols have been studied extensively for their beneficial effect against arthritis and the mechanisms involved in that process; for example, (1) green tea prepared from the dried leaves of *Camellia sinensis* is a commonly consumed beverage in many parts of the world. The polyphenolic compounds from green tea (PGT) possess anti-inflammatory and antiarthritic properties. PGT-induced reduction in clinical and histological features of arthritis was associated with a decrease in proinflammatory cytokine IL-17 and increase in anti-inflammatory cytokine IL-10 [44]. In another study using the CIA model of arthritis, PGT treatment reduced the expression of COX-2, IFN-*γ*, and TNF-*α* [43]. (2) Epigallocatechin-3-gallate (EGCG) is one of the most well-studied purified plant components against different diseases. EGCG inhibits IL-1-induced inducible nitric oxide synthase (iNOS), nitric oxide (NO), and JNK activity, all of which mediate cartilage degradation. It also suppresses IL-1-induced glycosaminoglycan release from cartilage via inhibiting ADAMTS (A disintegrin and metalloproteinase with thrombospondin motifs), MMP-1, and MMP-13 in chondrocytes. EGCG reduces osteoclast formation by inhibiting osteoblast differentiation without affecting their viability and proliferation [142]. Osteoclast-specific NFATc1 and bone resorption associated with RA are also suppressed. EGCG also inhibits RANKL-induced activation of JNK and NF*κ*B pathways, thereby suppressing the expression of c-Fos and NFATc1 in osteoclast precursors [143]. In another study, combination therapy of methotrexate and EGCG inhibited arthritis progression as evidenced by histopathology and radiographical examination [6]. Furthermore, this combination suppressed the expression of TNF-*α* and IL-6 in the joints of rats with AA [6]. (3) Grape seed proanthocyanidin extract (GSPE), an antioxidant derived from grape seeds, showed antiarthritic effect as evident from suppression of clinical signs of arthritis and IL-17 response along with increased Foxp3-expressing CD4^+^ regulatory T cells [45]. (4) Oligomeric procyanidins (HOPC) isolated from Jatoba, a South American herb, ameliorated arthritic inflammation and joint pathology in mice with CIA [42].

 (5) Resveratrol, a polyphenolic compound derived from grapes, suppressed swelling and bone erosion in the paws of mice with CIA [18]. This effect was associated with reduced serum levels of proinflammatory cytokines including IL-17 and reduced numbers of Th17 cells [18]. The antiarthritic effect of resveratrol also involves suppression of IL-1*β*, ROS, PGE2, and MMPs, and enhancement of proteoglycan synthesis and chondrocyte proliferation in vitro. (6) Curcumin, a principle component of turmeric, possesses anti-inflammatory and antiarthritic activity. Curcumin treatment downregulated clinical arthritis score, proliferation of splenic T cells, expression of TNF-*α* and IL-1*β* in the ankle joint, and serum IgG2a levels [144]. In addition, curcumin ameliorated the NF-*κ*B transcriptional activity in FLS and inhibited the production of PGE(2), COX-2 and MMP [144]. A randomized, pilot study conducted to assess the efficacy and safety of curcumin in RA patients revealed an improvement in overall disease activity score (DAS) and American College of Rheumatology (ACR) scores, and the treatment was safe without any significant adverse events [145]. (7) Capsaicin treatment suppressed bone erosion and trabecular damage in osteoarthritis in rats [34]. (8) Ferulic acid isolated from corn germ promotes bone remodeling and prevents bone loss in ovariectomized rats [36]. (9) Oleuropein aglycone, an olive oil-derived compound, improved clinical and histological features of arthritis in the joints of mice with CIA [37]. (10) Quercetin isolated from onion has a bone damage-protective attribute [38]. It inhibited RANKL-induced osteoclast differentiation and RANKL-stimulated osteoclast-related genes in rats with ovariectomy-induced bone loss [38]. (11) Silibinin, a major active constituent of silymarin, inhibits osteoclast formation by attenuating the downstream signaling cascades associated with RANKL and TNF-*α*, such as osteoclast-associated receptor (OSCAR), NFATc1, Cat K, and MMP-9 [9]. (12) Rosmarinic acid is an active component of *Plectranthus amboinicus. *It inhibited RANKL-induced formation of TRAP-positive multinucleated cells and suppressed NF-*κ*B activation and NFATc1 nuclear translocation in the CIA model [9].

## 9. Concluding Remarks

It is increasingly been realized that inflammation and bone damage are generally linked in arthritis and various other disorders. A better understanding of the shared processes would help define the molecules and pathways that can serve as targets for effective antiarthritic therapy. In parallel, it is essential to search for newer therapeutic agents that are both effective but safe. In this regard, herbal products may offer promising alternatives or adjuncts to conventional antiarthritic agents. In this paper, we have elaborated upon both these important aspects of immune pathology and herbal therapy of arthritis.

## Figures and Tables

**Table 1:** Natural products that have antiarthritic/bone protective property.

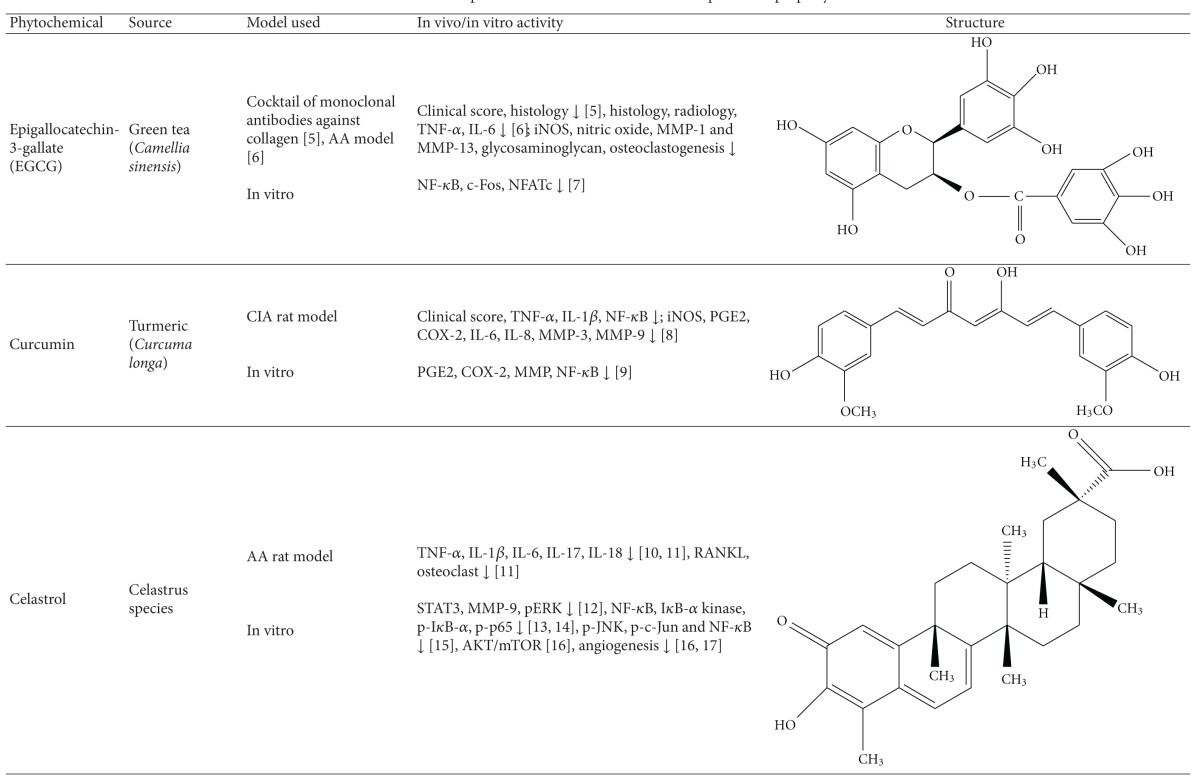 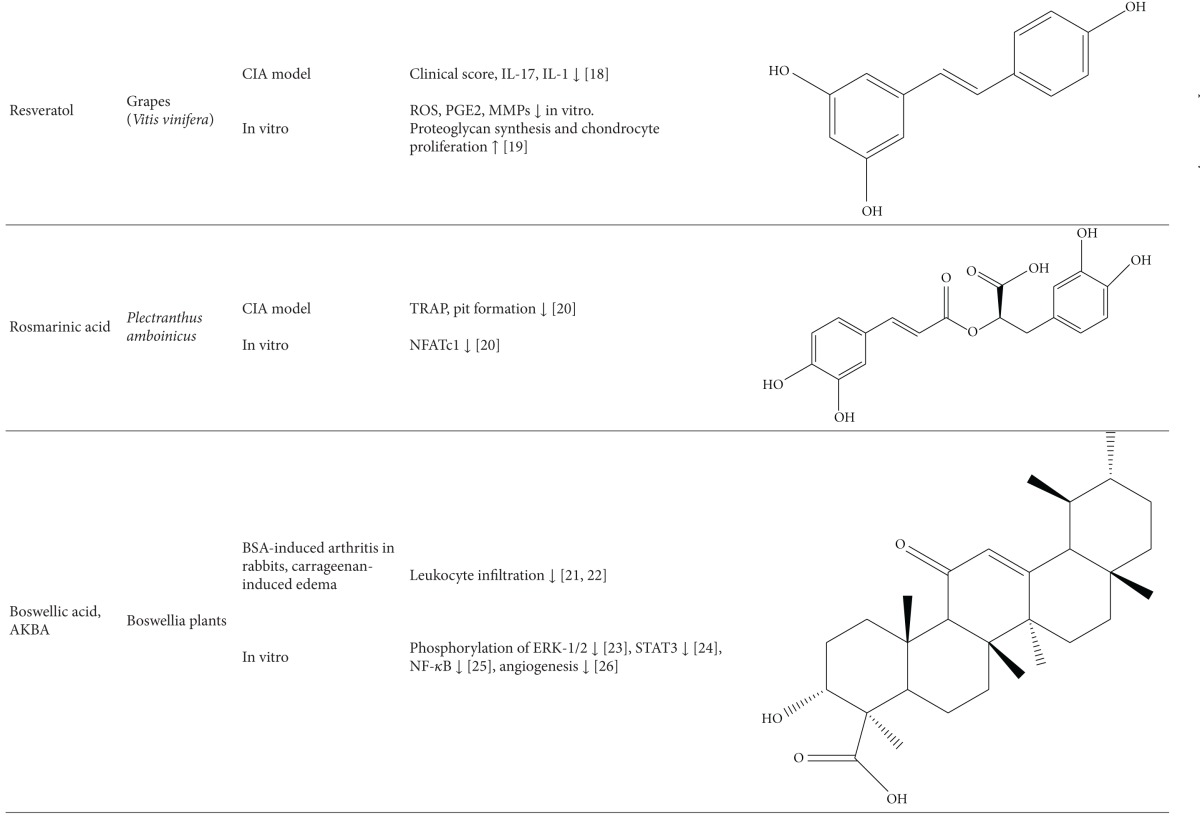 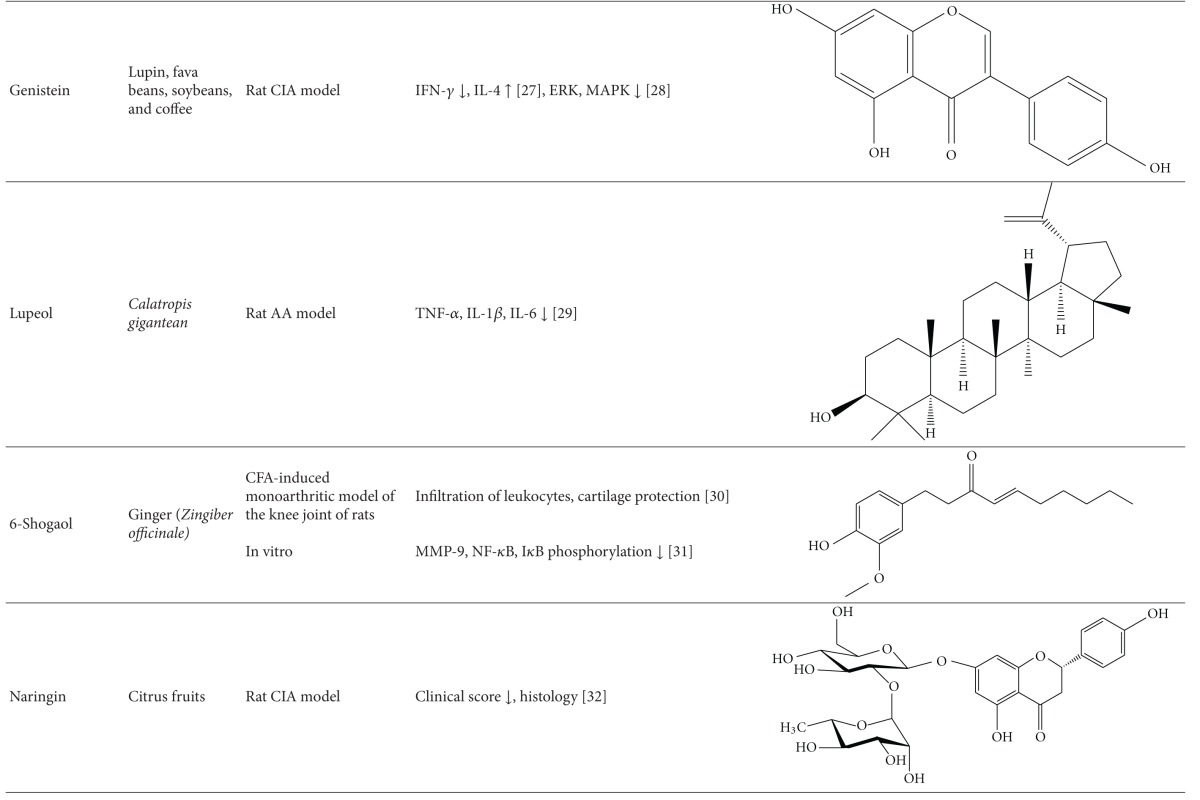 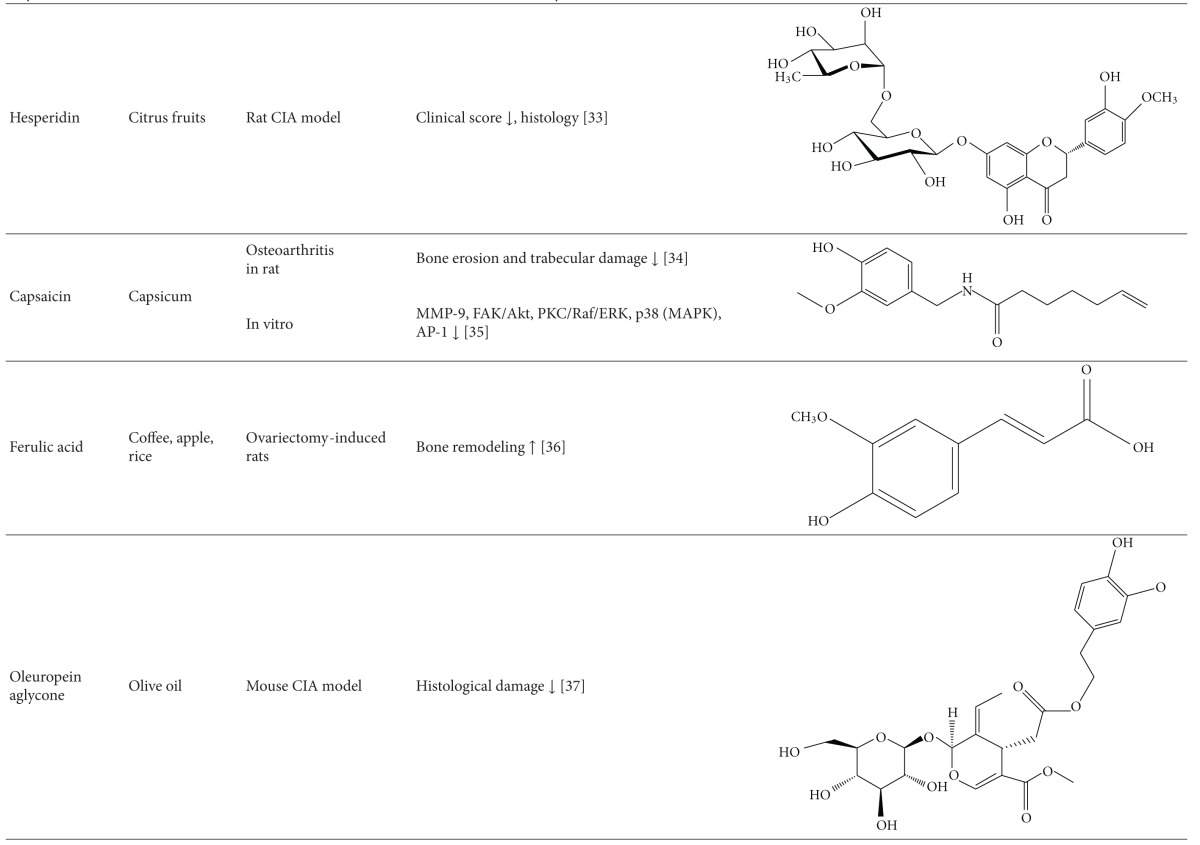 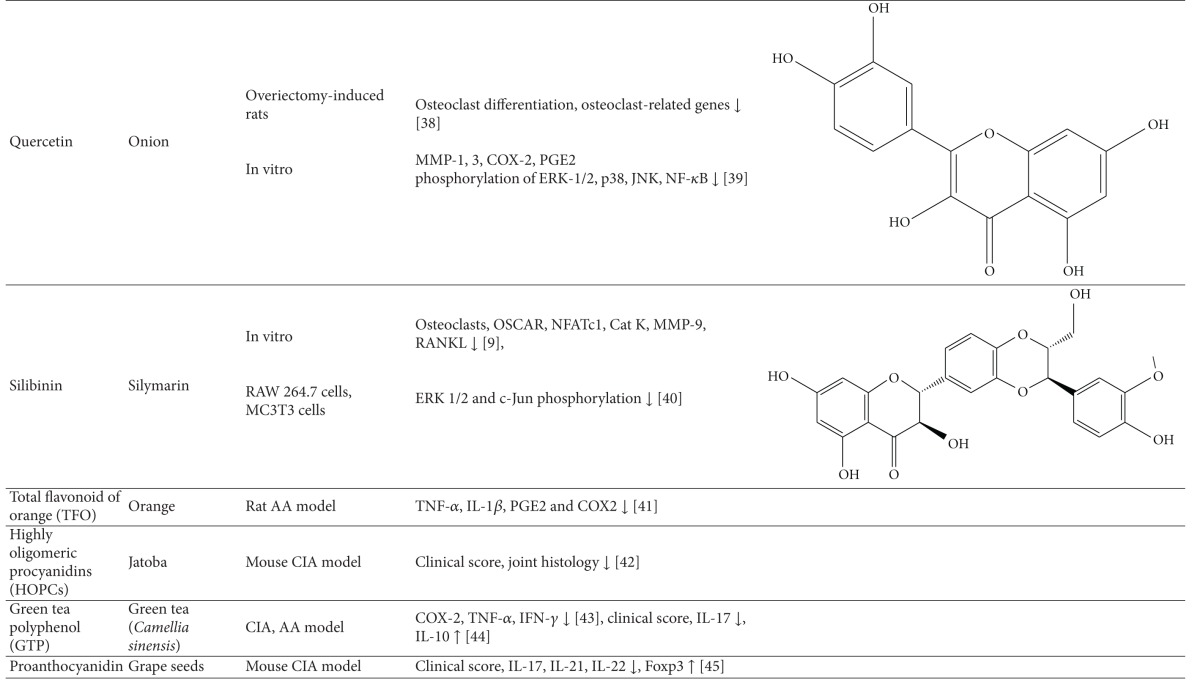

## Abbreviations

AA: Adjuvant arthritis
Bhsp65: Mycobacterial heat-shock protein 65
CatK: Cathepsin K
CIA: Collagen-induced arthritis
DCs: Dendritic cells
FLS: Fibroblast-like synoviocytes
Cbfa: Corebinding factor alpha
GM-CSF: Granulocyte-macrophage colony-stimulating factor
IFN-*γ*: Interferon gamma
IGF: Insulin-like growth factor
JNK: c-Jun amino-terminal kinase
LNCs: Lymph node cells
M-CSF: Macrophage colony-stimulating factor
MAPK: Mitogen-activated protein kinase
MMPs: Matrix metalloproteases
Mtb: *My* *co* *ba* *ct* *er* *iu* *m* *tuberculosis*
NFAT: Nuclear factor of activated T-cells
NF-*κ*B: Nuclear factor-kappa B
OCN: Osteocalcin
OPN: Osteopontin
OPG: Osteoprotegerin
PG: Prostaglandin
PTH: Parathyroid hormone
PKB: Protein kinase B
RA: Rheumatoid arthritis
RANKL: Receptor activator of NF-*κ*B ligand
ROS: Reactive oxygen species
Runx: Runt-related transcription factor
SF: Synovial fluid
SICs: Synovial infiltrating cells
SACs: Spleen adherent cells
Th17: T helper 17
TIMP: Tissue inhibitor of metalloproteinase
TNF: Tumor necrosis factor-*α*
TGF: Transforming growth factor
TRAFs: TNFR-associated factors
TRAP: Tartrate-resistant acid phosphatase.
